# 

**Published:** 1864-06

**Authors:** 


### BOOKS RECEIVED.

*The Principles and Practice of Obstetrics: Illustrated with One Hundred and Fifty-Nine Lithographic Figures, from Original Photographs and with numerous Wood Cuts. By* HUGH L. HODGE, M. D., *Emeritus Prof. of Obstetrics and Diseases of Women and Children, in the University of Pennsylvania; lately one of the Physicians to the Lying-in Department of the Pennsylvania Hospital; lately one of the Physicians to the Philadelphia Almshouse Hospital; Consulting Physician to the Philadelphia Dispensary; Fellow of the College of Physicians of Philadelphia; Member of the American Philosophical Society, etc., Author of a treatise on "The Peculiar Diseases of Women." Philadelphia:* BLANCHARD & LEA—1864.

*Medical Diagnosis, with Special reference to Practical Medicine; a Guide to the knowledge and discrimination of Diseases. By* J. M. DA COSTA, M. D, *Lecturer on Clinical Medicine, and Physician to the Philadelphia Hospital; Fellow to the College of Physicians of Philadelphia; Corresponding Member of the New York Pathological Society, etc., etc., Illustrated with Engravings on Wood. Philadelphia:* J. B. LIPPINCOTT & Co—1864.

*The Pathology and Treatment of Venereal Diseases, including the results of recent Investigations upon the Subject. By* FREEMAN J. BUMSTEAD, M. D., *Lecturer on Venereal Diseases at the College of Physicians and Surgeons, New York; late Surgeon to St. Luke's Hospital; Surgeon to the New York Eye and Ear Infirmary. A new and Revised Edition, with Illustrations. Philadelphia:* BLANCHARD & LEA—1864.

*A Manual of the Practice of Medicine. By* THOMAS HAWKES TANNER, M. D., F. L. S., *Member of the Royal College of Physicians; Assistant Physician for the Diseases of Women and Childrea to King's College Hospital, etc., etc. From the last London Edition, Enlarged and Improved. Philadelphia:* LINDSAY & BLAKISTON—1864.

*On Rheumatism, Rheumatic Gout and Sciatica, their Pathology, Symptoms and Treatment. By* HENRY WILLIAM FULLER, M. D,, *Cantab., Fellow of the Royal College of Physicians, London; Physician to St. George's Hospital, etc., etc. From the last London Edition. Philadelphia:* LINDSAY & BLAKISTON—1864.

*A Treatise on the Chronic Inflammation and Displacements of the Unimpregnated Uterus. By* WM. H. BYFORD, A. M. M. D., *Professor of Obstetrics, etc., etc., Chicago Medical College, Medical Department Lind University. Philadelphia:* LINDSAY & BLAKISTON—1864.

*Lectures on Orthopædic Surgery. Delivered at the Brooklyn Medical and Surgical Institute. with numerous Illustrations. By* LOUIS BAUER, M. D., M. R. C. S., Eng., *Professor of Anatomy and Clinical Surgery; Licentiate of the New York State Medical Society; Member of the New York Pathological Society, of the American Medical Association; Corresponding Fellow of the London Medical Society; Health Officer of the City of Brooklgn, etc. (Re-printed from the Philadelphia Medical and Surgical Reporter.) Philadelphia:* LINDSAY & BLAKISTON—1864.

*Lectures on Medical Education, or on the Proper Method of studying Medicine. By* Samuel Chew, M. D., *Professor of the Practice and Principles of Medicine, and of Clinical Medicine in the University of Maryland. Philadelphia:* Lindsay & Blakiston—1864.

*Annual Announcement of the Medical Department of the University of Buffalo, for the Session of* 1864–5.

*A new Opthalmoscope, for Photographing the Posterior Internal surface of the living Eye; with an outline of the Theory of the ordinary Opthalmoscope. By* A. M. Rosebrugh, M. D. *Read before the Canadian Institute, January* 16*th*, 1864.

